# Selenium-SelK-GPX4 axis protects nucleus pulposus cells against mechanical overloading-induced ferroptosis and attenuates senescence of intervertebral disc

**DOI:** 10.1007/s00018-023-05067-1

**Published:** 2024-01-22

**Authors:** Chunwang Jia, Ziqian Xiang, Pengfei Zhang, Long Liu, Xuetao Zhu, Ruixuan Yu, Zhicheng Liu, Shaoyi Wang, Kaiwen Liu, Zihao Wang, Krasimir Vasilev, Shuanhu Zhou, Ziwen Geng, Xinyu Liu, Yunpeng Zhao, Yuan Gao, Lei Cheng, Yuhua Li

**Affiliations:** 1 Department of Orthopaedics, Cheeloo College of Medicine, Qilu Hospital, Shandong University, 107 Wenhuaxi Road, Jinan, 250012 Shandong People’s Republic of China; 2 Department of Pathology, Cheeloo College of Medicine, Qilu Hospital, Shandong University, Jinan, 250012 Shandong People’s Republic of China; 3 Academic Unit of STEM, University of South Australia, Mawson Lakes, Adelaide, SA 5095 Australia; 4 College of Medicine and Public Health, Flinders University, Bedford Park, SA 5042 Australia; 5 Department of Orthopedic Surgery, Brigham and Women’s Hospital, Harvard Medical School, Boston, MA USA; 6 Qilu Institute of Technology, Jinan, 250200 Shandong People’s Republic of China

**Keywords:** Selenium, SelK, GPX4, Mechanical stress, Endoplasmic reticulum stress, Ferroptosis

## Abstract

Intervertebral disc degeneration (IVDD) is one of the most prevalent spinal degenerative disorders and imposes places heavy medical and economic burdens on individuals and society. Mechanical overloading applied to the intervertebral disc (IVD) has been widely recognized as an important cause of IVDD. Mechanical overloading-induced chondrocyte ferroptosis was reported, but the potential association between ferroptosis and mechanical overloading remains to be illustrated in nucleus pulposus (NP) cells. In this study, we discovered that excessive mechanical loading induced ferroptosis and endoplasmic reticulum (ER) stress, which were detected by mitochondria and associated markers, by increasing the intracellular free Ca^2+^ level through the Piezo1 ion channel localized on the plasma membrane and ER membrane in NP cells. Besides, we proposed that intracellular free Ca^2+^ level elevation and the activation of ER stress are positive feedback processes that promote each other, consistent with the results that the level of ER stress in coccygeal discs of aged Piezo1-CKO mice were significantly lower than that of aged WT mice. Then, we confirmed that selenium supplementation decreased intracellular free Ca^2+^ level by mitigating ER stress through upregulating Selenoprotein K (SelK) expression. Besides, ferroptosis caused by the impaired production and function of Glutathione peroxidase 4 (GPX4) due to mechanical overloading-induced calcium overload could be improved by selenium supplementation through Se-GPX4 axis and Se-SelK axis in vivo and in vitro, eventually presenting the stabilization of the extracellular matrix (ECM). Our findings reveal the important role of ferroptosis in mechanical overloading-induced IVDD, and selenium supplementation promotes significance to attenuate ferroptosis and thus alleviates IVDD, which might provide insights into potential therapeutic interventions for IVDD.

**Supplementary Information:**

The online version contains supplementary material available at 10.1007/s00018-023-05067-1.

## Introduction

Mechanical overloading is strongly associated with various damage in different cell types [1–3]. IVDD is one of the most prevalent spinal degenerative disorders and places heavy medical and economic burdens on individuals and society [4]. Mechanical overloading applied to IVD due to prolonged standing and obesity has been widely recognized as an important cause of IVDD, and NP bears 75 percent of the pressure [5, 6]. However, the specific mechanism of the induction of IVDD by mechanical overloading has not been fully elucidated.

GPX4 is the major lipid peroxidation scavenger and plays a critical regulatory role in ferroptosis [7]. It is reported that enhanced calcium influx due to the Piezo1 ion channel activated by mechanical overloading induces GPX4-regulated ferroptosis [8]. Besides, as a crucial intracellular calcium pool, ER plays an important role in regulating intracellular free calcium [9]. The activation of Piezo1 localized at the ER membrane has been found to increase intracellular free calcium by inducing calcium release from ER [10]. However, it remains to be determined whether mechanical overloading would simultaneously activate Piezo1 ion channel localized on plasma membrane and ER membrane to elevated intracellular free Ca^2+^ levels in NP cells, which leads to GPX4-regulated ferroptosis.

Activation of ER stress is thought to exacerbate cell ferroptosis in injuries of kidney and lung but the mechanism by which ER stress regulates ferroptosis remains unclear [11, 12]. SelK is an ER-resident protein, which has been shown to be involved in ER stress and cellular oxidation resistance [13]. Selenium is an essential micronutrient with protective effects against oxidative stress [14]. The biological role of selenium is mainly exerted by its incorporation into selenoproteins as selenocysteine [15]. It’s widely accepted that selenium supplementation could upregulate the expression of various selenoproteins, and the protective effect from ferroptosis of the Selenium–GPX4 axis was demonstrated in follicular helper T cells [16, 17]. However, there have been no reports of whether selenium supplementation could attenuate ferroptosis by upregulating SelK and GPX4 in NP cells.

In this study, we plan to explore whether mechanical overloading could obstruct GPX4 production by activating Piezo1 ion channel localized on plasma membrane and endoplasmic reticulum membrane, which then leads to ferroptosis in NP cells. We will determine the factors promoting ER stress and the specific role of ER stress in ferroptosis during the above process. In addition, we will verify whether selenium supplementation could attenuate mechanical overloading-induced ferroptosis of NP cells by upregulating GPX4 and SelK.

## Results

### Excessive mechanical loading induces ferroptosis and ER stress in NP cells

**Fig. 1:**
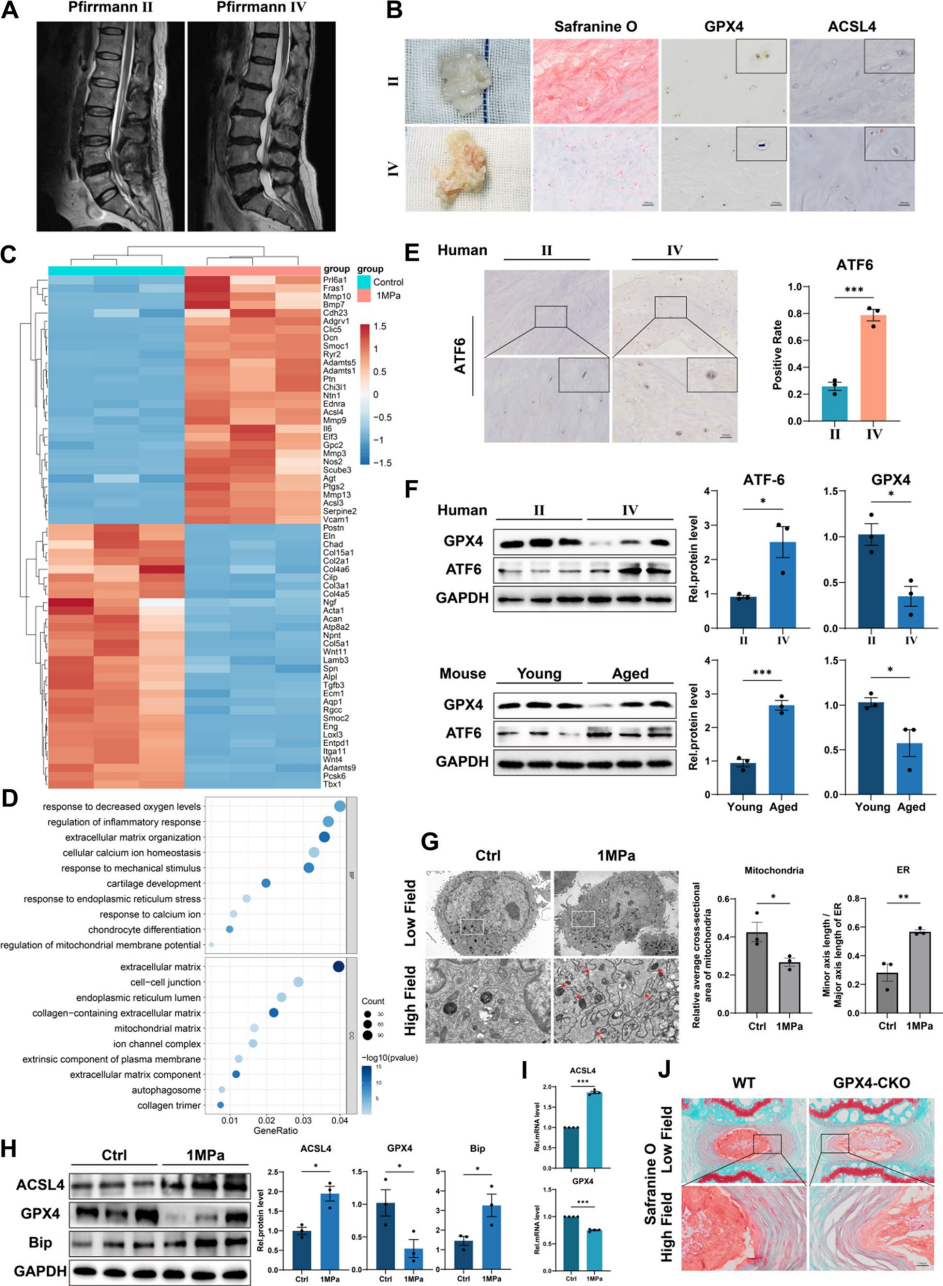
Excessive mechanical loading induces ferroptosis and ER stress in NP cells. **A** Typical human MR T2-weighted images of grade II (left panel) and grade IV (right panel) IVD. **B** General view of NP from donors who accepted NP discectomy surgery. The Safranine O-Fast Green staining of NP from human degenerated disc. The IHC staining of GPX4 and ACSL4 in human NP sections. Scale bar: 100 μm. **C** Microarray heatmap of rat NP cells from the control group and 1 MPa group (n = 3 for each group). **D** GO enrichment analysis of the indicated groups. **E** The IHC staining of ATF6 in human NP sections and the quantitative analysis. Scale bar: 100 μm. **F** The Western blotting analysis for GPX4 and ATF6 of NP tissues from humans and mice. **G** Representative TEM images and quantitative analysis of rat NP cells from the control group and 1 MPa group. Arrows show shrunken mitochondria and swollen ER. Scale bars: 5 μm (Low field), 1 μm (High field). **H** The Western blotting for ACSL4, GPX4, and Bip and quantification analysis (n = 3 for each group). **I** The qPCR of ACSL4, GPX4 (n = 4 for each group). **J** The Safranine O-Fast Green staining of coccygeal discs of WT mice and GPX4-CKO mice. Scale bar: 50 μm. Results were shown as mean ± SEM. *p < 0.05, ***p < 0.001

To investigate the changes of ferroptosis-related indicators in NP tissue during IVDD, we collected tissue samples from patients who accepted lumbar discectomy surgery because of lumbar disc herniation. The Pfirrmann grade of degenerated discs was evaluated according to signal intensity in T2 Weight-Images of Magnetic Resonance (Fig. 1A) [18]. Specifically, NP tissues of late-stage degenerated discs showed atrophic volume and decreased elasticity compared to those of earlier stages (Fig. 1B). Then Safranine O staining and immunohistochemistry (IHC) for GPX4 and ACSL4 were performed. Our results showed that the expression level of the ferroptosis regulator GPX4 was reduced in degenerative NP tissues (grade IV), but the ferroptosis biomarker ACSL4 expression level was increased (Fig. 1B). Excessive mechanical loading is important in IVDD, which is associated with ferroptosis in chondrocytes [19]. The NP cells are similar to chondrocytes in morphology and function [20]. To verify the relationship between excessive mechanical loading and ferroptosis in NP, primary rat NP cells were isolated and cultured under 1 MPa of mechanical stress at 1 Hz for 1 h. Thereafter, the NP cells were cultured for another 24 h, and microarray assay was performed. Microarray results indicated the ECM metabolism disorder and an increase of the ferroptosis biomarker ACSL4 in NP cells stimulated with mechanical stress compared with the control group (Fig. 1C). Interestingly, Gene Ontology (GO) analysis revealed the differentially expressed proteins were highly relevant to cellular calcium ion homeostasis and response to ER stress, and ER emerged as one of the most dramatically enriched cellular component terms (Fig. 1D). Moreover, the total mRNA was extracted at 3 h, 6 h and 12 h after stress apply and analyzed by qPCR to confirm excessive mechanical loading leading to ER stress and ferroptosis (Figure S1). Then, IHC for the ER stress biomarker ATF6 was performed, and we found a significant increase of ATF6 in grade IV NP tissues compared to grade II (Fig. 1E). Besides, Western blotting analysis of NP tissues from humans revealed a reduction in GPX4 levels but an increase in ATF6 levels in degenerative NP tissues (grade IV), Which is consistent with the Western blotting analysis of NP tissues from young mice coccygeal discs (8- to 12-weeks old, n = 5) as well as aged mice coccygeal discs (12- to 18-months old, n = 5) (Fig. 1F). Moreover, transmission electron microscopy (TEM) was performed to investigate the effect of mechanical stimuli on NP cells. Compared to the control group, the ER showed an abnormal swelling in structure, and the mitochondria exhibited ferroptosis-related changes like thickened mitochondrial membranes and shrinkage of mitochondria in the mechanical overloading group, which were quantified by Minor axis length/Major axis length of ER and relative average cross-sectional area of mitochondria (Fig. 1G) [21–24]. Then, Western blotting and qPCR revowed that mechanical stimuli decreased GPX4 level, increased ACSL4 level and the ER stress marker Bip level (Fig. 1H, I). In addition, tamoxifen-inducible homozygous GPX4 conditional knockout mice (Col2a1-CreERT, GPX4^flox/flox^) were established by mating Col2a1-CreERT mice with GPX4^flox/flox^ mice to elucidate the physiological roles of endogenous GPX4 in IVD. And the safranine-O staining of coccygeal discs of WT mice and GPX4-CKO mice (6- to 8-months old, n = 5) indicated GPX4 was important in IVD physiology (Fig. 1J).

### Excessive mechanical loading induces ferroptosis and ER stress by increasing the intracellular free Ca^2+^ level through the Piezo1 ion channel localized on the plasma membrane and ER membrane

**Fig. 2:**
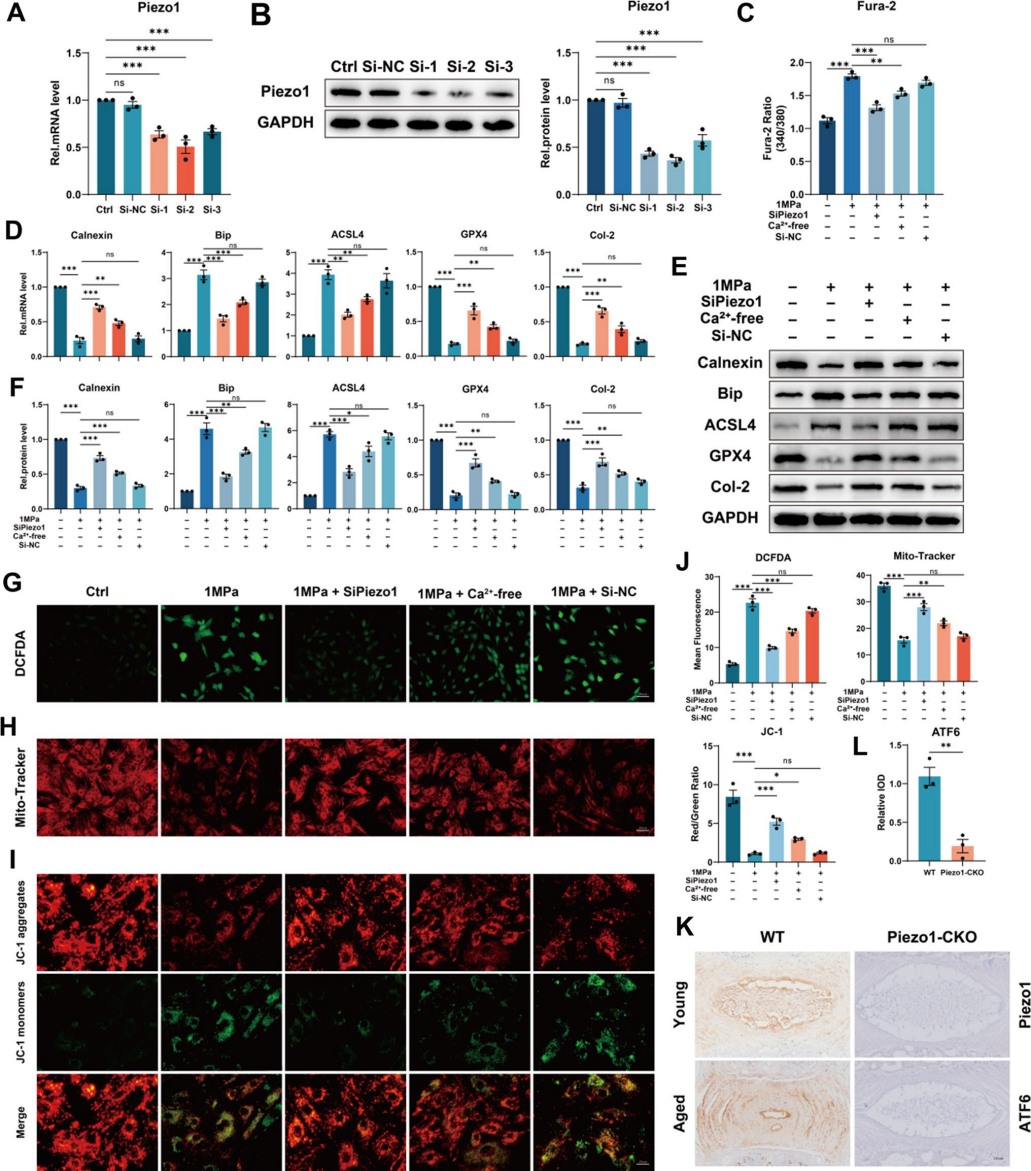
Excessive mechanical loading induces ferroptosis and ER stress by increasing the intracellular free Ca^2+^ level through the Piezo1 ion channel localized on the plasma membrane and ER membrane. **A** The qPCR of Piezo1 of the groups in this Figure (n = 3 for each group). **B** The Western blotting analysis for Piezo1 (n = 3 for each group). **C** The Fura-2 AM to detect intracellular free Ca^2+^ level (n = 3 for each group). **D** The qPCR of Calnexin, Bip, ACSL4, GPX4 and Col-2 (n = 3 for each group). **E** The Western blotting analysis for Calnexin, Bip, ACSL4, GPX4 and Col-2. **F** Quantification of Western blotting analysis (n = 3 for each group). **G**–**I** DCFDA assays for ROS level and Mito-Tracker and JC-1 for mitochondrial membrane potential. Scale bar: 50 or 25 μm. **J** Quantitative analysis for DCFDA, Mito-Tracker and JC-1 (n = 3 for each group). **K** The IHC staining of Piezo1 and ATF6 for WT and Piezo1-CKO mice. Scale bar: 250 μm. **L** Quantitative analysis for the IHC staining of ATF6 (n = 3 for each group). Results were shown as mean ± SEM. *ns* not significant, *p < 0.05, **p < 0.01, ***p < 0.001

Increased intracellular free Ca^2+^ level impairs the function of GPX4 and leads to the destruction of mitochondria and ferroptosis [25, 26]. The Piezo1 ion channel is activated by mechanical stimuli, which facilitates extracellular calcium influx, and upregulation of Piezo1 enhances the intracellular calcium release from the endoplasmic reticulum [8, 10, 27]. Besides, intracellular free Ca^2+^ imbalance induces ER stress [28]. Here, we used SiPiezo1 and calcium-free medium to investigate the association between ferroptosis, ER stress and mechanical overloading, where SiPiezo1 was used to knock down Piezo1 (Fig. 2A, B), and the calcium-free medium was used to remove extracellular Ca^2+^. To confirm the involvement of Ca^2+^, the cells were pretreated with intracellular Ca^2+^ chelator, BAPTA-AM (10 μM) for 2 h prior to stress apply.The intracellular free Ca^2+^ level was detected by Fura-2 AM and Calnexin, an ER-resident protein with high capacity for Ca^2+^ binding, function of which depends on the calcium concentration in the ER and ATP content [29] and which is susceptible to be broken down by protease in cases of low calcium [30], was analyzed to observed the ER Ca^2+^ level [31]. Interestingly, removing extracellular Ca^2+^ alleviated the increase of intracellular free Ca^2+^ level induced by mechanical stimuli, while knock down of Piezo1 has more potent effects (Fig. 2C). On the other hand, the Calnexin level was inversely proportional to the intracellular free Ca^2+^ level, which indicated that excessive mechanical loading increased the intracellular free Ca^2+^ level through the Piezo1 ion channel localized on the plasma membrane and ER membrane (Fig. 2C–F). Similarly, additional knock down of Piezo1 reduced the rise of ER stress biomarker Bip due to mechanical stimulation, an effect better than merely removing extracellular Ca^2+^, which was detected by qPCR and Western blotting (Fig. 2D–F). Correspondingly, except for the control group, GPX4 expression in the SiPiezo1 group was the highest, followed by the Ca^2+^-free group and ACSL4 expression was lowest (Fig. 2D–F). In the present study, mechanical overloading increased Reactive oxygen species (ROS) production and impaired the membrane potential of mitochondria in NP cells, while both knock down of Piezo1 and removal of extracellular Ca^2+^ reversed these changes, but the former was more effective (Fig. 2G–J). Interestingly, knock down of Piezo1 upregulated the decreased level of Col-2 induced by mechanical stimuli while removing extracellular Ca^2+^ showed weaker effects (Fig. 2D–F). Moreover, Piezo1-CKO mice were established by mating Col2a1-CreERT mice with Piezo1^flox/flox^ mice to elucidate the roles of Piezo1 in ER-Stress. And immunohistochemistry of ATF6 indicated the level of ER stress in coccygeal discs of aged Piezo1-CKO mice were significantly lower than that of aged WT mice (Fig. 2K, L). Besides, PMCA1 is one type of PMCA widely found in various cells, it has been reported that there is time-dependent reduced expression of PMCA1 in stretched myoblasts [32]. In our study, mechanical overloading reduced PMCA1 expression (Fig. S3), consistent with the observation of elevation of intracellular free Ca^2+^ level. Although the reduced PMCA1 expression could be sufficient to explain the elevation of intracellular Ca^2+^ elevation regardless of Piezo1. After Piezo1 was knocked down by SiPiezo1, the elevation of intracellular free Ca2+ level was significantly reversed, suggesting the role of Piezo1. So PMCA1 does mediated part of the phenotype in our study and the specific mechanism of PMCA1 changes remains to be further explored. In summary, mechanical overloading induces ER stress and ferroptosis by increasing the intracellular free Ca^2+^ level through the Piezo1 ion channel localized on the plasma membrane and ER membrane in NP cells.

### Suppression of ER stress attenuates mechanical overloading-induced ferroptosis by alleviating Ca^2+^ outflow from ER

**Fig. 3:**
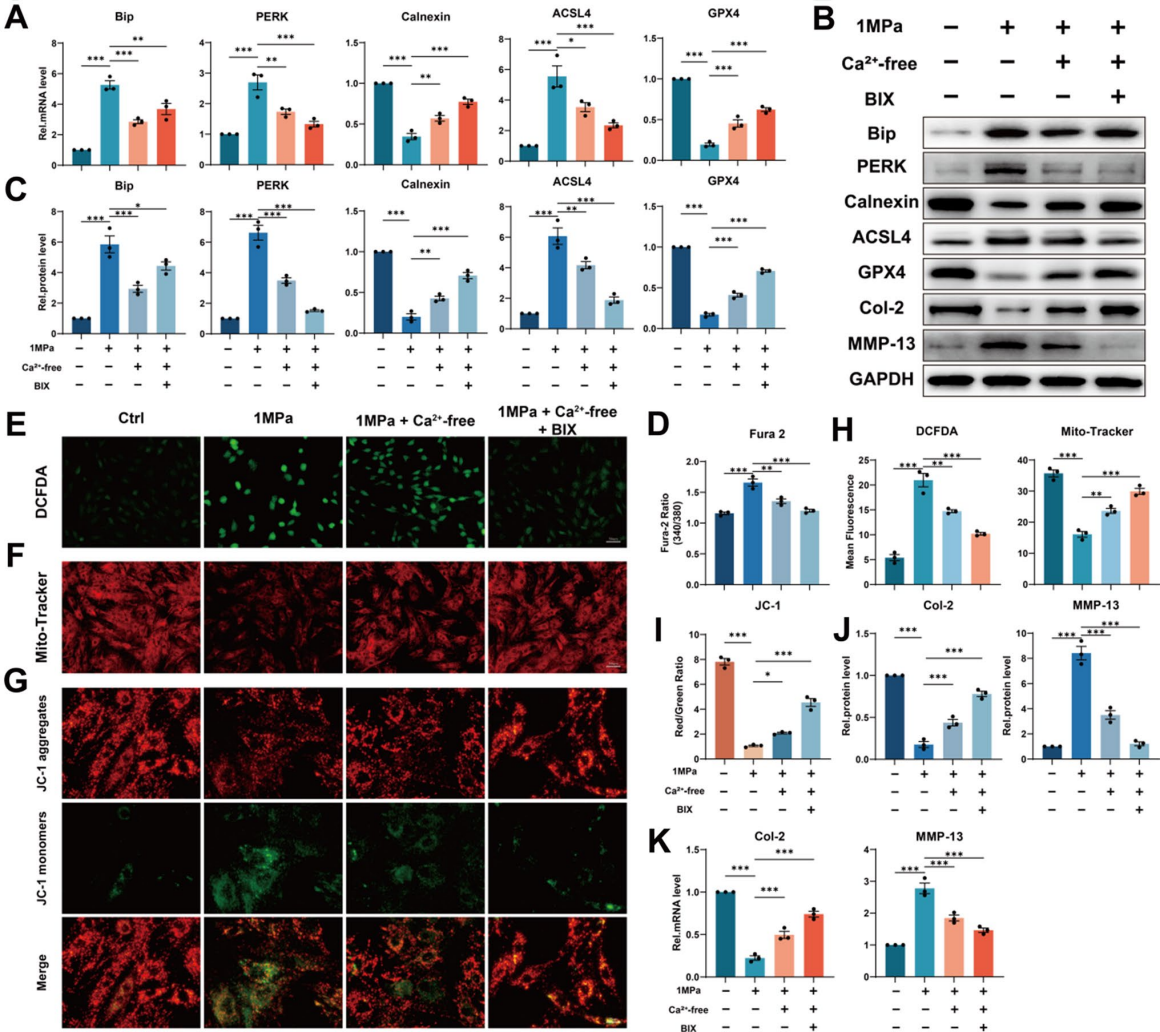
Suppression of ER stress attenuates mechanical overloading-induced ferroptosis by alleviating Ca^2+^ outflow from ER. **A** The qPCR of Bip, PERK, Calnexin, ACSL4 and GPX4 of the groups in this Figure (n = 3 for each group). **B** The Western blotting analysis for Bip, PERK, Calnexin, ACSL4, GPX4, Col-2 and MMP-13. **C** Quantification of Western blotting analysis (n = 3 for each group). **D** The Fura-2 AM to detect intracellular free Ca^2+^ level (n = 3 for each group). **E**–**G** DCFDA assays for ROS level and Mito-Tracker and JC-1 for mitochondrial membrane potential. Scale bar: 50 or 25 μm. **H**–**I** Quantitative analysis for DCFDA, Mito-Tracker, and JC-1 (n = 3 for each group). **J** The qPCR of Col-2 and MMP-13 of the groups in this Figure (n = 3 for each group). **K** Quantification of Western blotting analysis(n = 3 for each group). Results were shown as mean ± SEM. *p < 0.05, **p < 0.01, ***p < 0.001

It’s reported that inhibition of ER stress could alleviate ferroptosis in colonic epithelial cells [33] and we earlier found that Ca^2+^ may be the key nodes between ER stress and ferroptosis. Here, the Ca^2+^-free medium and the ER stress inhibitor Bip inducer X (BIX) were used to explore further the association between ER stress, ferroptosis and intracellular free Ca^2+^. Also before stress apply, the cells were pretreated with BAPTA-AM (10 μM) for 2 h. Our data showed that, additional treatment with BIX upregulated the level of Bip, which improved ER stress and further reduced the level of the ER stress biomarker PERK as compared to the Ca^2+^-free group (Fig. 3A–C). Surprisingly, intracellular free Ca^2+^ level was reduced after inhibition of ER stress during the above process (Fig. 3D). Correspondingly, the Calnexin level was observed to be inversely proportional to intracellular free Ca^2+^ level (Fig. 3A–C). Then qPCR and Western blotting revealed the positive proportional trend of the level of ACSL4 and the opposite trend of the level of GPX4 to intracellular free Ca^2+^ level in NP cells (Fig. 3A–C). Besides, suppression of ER stress further decreased the elevated ROS synthesis and alleviated the mitochondrial dysfunction (Fig. 3E–I). Expectedly, qPCR and Western blotting revealed suppression of ER stress reduced the production of ECM catabolic biomarker MMP-13 and promoted the secretion of ECM anabolic biomarker Col-2 (Fig. 3B, J–K). In conclusion, we came to that suppression of ER stress attenuates mechanical overloading-induced ferroptosis by alleviating Ca^2+^ outflow from ER.

### Selenium supplementation protects NP cells from ferroptosis by enhancing GPX4 expression

**Fig. 4:**
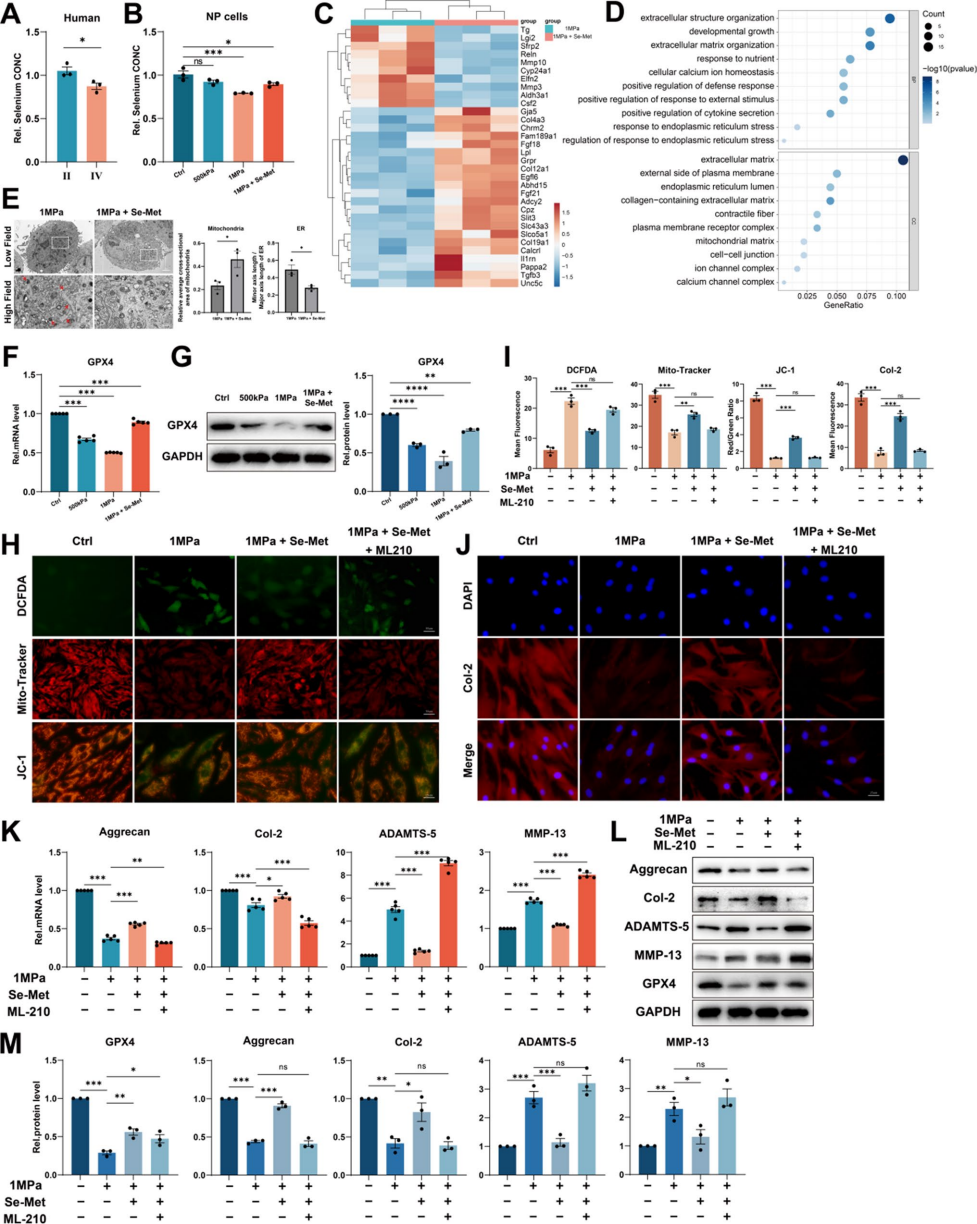
Selenium supplementation protects NP cells from ferroptosis by enhancing GPX4 expression. **A** The concentration of selenium in NP tissues from humans (n = 3 for each group). **B** The concentration of selenium of NP cells (n = 3 for each group). **C** Microarray heatmap of rat NP cells from the 1 MPa group, and 1 MPa + Se-Met group (n = 3 for each group). **D** GO enrichment analysis of the indicated groups. **E** Representative TEM images and quantitative analysis of rat NP cells from 1 MPa group and 1 MPa + Se-Met group. Arrows show shrunken mitochondria and swollen ER. Scale bars: 2 μm (Low field), 1 μm (High field). **F** The qPCR of GPX4 of the groups in this Figure (n = 5 for each group). **G** The Western blotting analysis for GPX4 (n = 3 for each group). **H** DCFDA assays for ROS level and Mito-Tracker and JC-1 for mitochondrial membrane potential. Scale bar: 50 or 25 μm. **I** Quantitative analysis of mean fluorescence (n = 3 for each group). **J** The immunofluorescence of Col-2. Scale bar: 25 μm. **K** The qPCR of Aggrecan, Col-2, ADAMTS-5, and MMP-13 of the groups in this Figure (n = 5 for each group). **L** The Western blotting analysis for Aggrecan, Col-2, ADAMTS-5, and MMP-13. **M** Quantification of Western blotting analysis (n = 3 for each group). Results were shown as mean ± SEM. *ns* not significant, *p < 0.05, **p < 0.01, ***p < 0.001

Selenium shows anti-inflammation, anti-oxidation, and anti-aging effects as an essential micronutrient [34, 35]. Selenium-GPX4 axis was proven to protect follicular helper T cells from ferroptosis [16]. We collected human NP tissues of different grades and examined the concentration of selenium and found the concentration of selenium was reduced in degenerative NP tissues (grade IV) (Fig. 4A). Moreover, primary rat NP cells were cultured under 500 kPa or 1 MPa of mechanical stress with or without Se-Met supplementation, and high strain loading reduced the concentration of selenium, but Se-Met supplementation reversed this change (Fig. 4B). Then microarray assay and GO analysis were performed, which indicated Se-Met supplementation improved the ECM metabolism disorder and promoted cell response to calcium ion and ER stress (Fig. 4C, D). TEM was also performed and suggested Se-Met supplementation improved swollen ER and shrunken mitochondria induced by mechanical overloading, which were quantified by Minor axis length/Major axis length of ER and relative average cross-sectional area of mitochondria (Fig. 4E). Then qPCR and Western blotting revealed that high strain loading reduced the GPX4 mRNA and protein level while Se-Met supplementation promoted GPX4 expression (Fig. 4F, G). As shown, Se-Met supplementation improved the elevated ROS synthesis and mitochondrial dysfunction induced by mechanical overloading but this phenomenon could no longer be observed when GPX4 inhibitor ML210 was additionally applied (Fig. 4H, I). The results of Immunofluorescence for Col-2 suggested Se-Met supplementation promoted the expression of Col-2 but ML210 restricted this effect (Fig. 4I, J). Then qPCR and Western blotting revealed that Se-Met supplementation reduced the production of ADAMTS-5 and MMP-13 and promoted the secretion of Aggrecan and Col-2 in response to mechanical overloading, while additional ML210 aggravated ECM metabolic disorder (Fig. 4K–M).

### Selenium supplementation mitigates ER stress through upregulating SELK expression

**Fig. 5:**
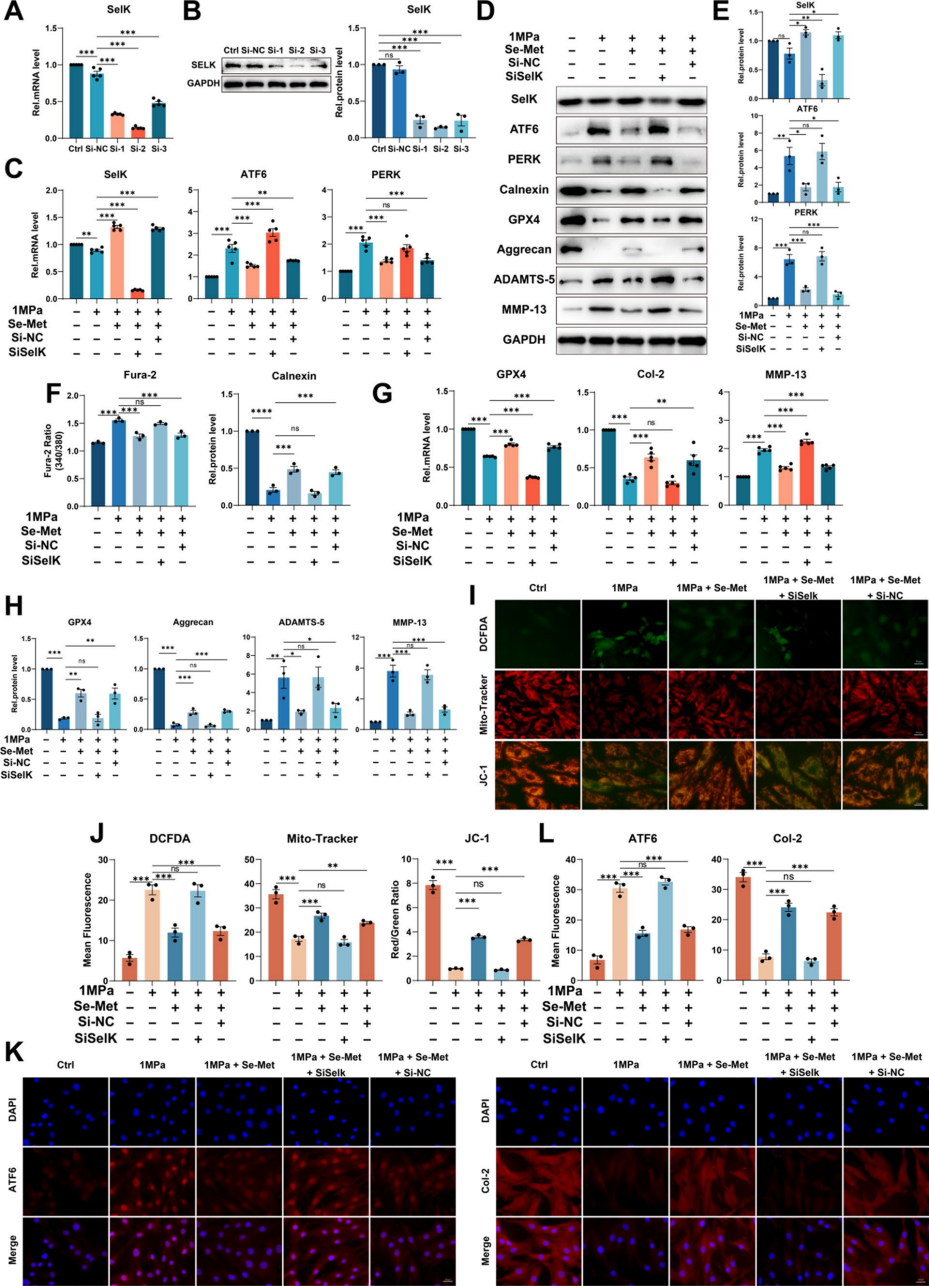
Selenium supplementation mitigates ER stress through upregulating SELK expression. **A** The qPCR of SelK of the groups in this Figure (n = 5 for each group). **B** The Western blotting analysis for SelK (n = 3 for each group). **C** The qPCR of SelK, ATF6 and PERK of the groups in this Figure (n = 5 for each group). **D** The Western blotting analysis for SelK, ATF6, PERK, Calnexin, GPX4, Aggrecan, ADAMTS-5 and MMP-13. **E** Quantification of Western blotting analysis for SelK, ATF6 and PERK (n = 3 for each group). **F** The Fura-2 AM to detect intracellular free Ca^2+^ level (n = 3 for each group). **G** The qPCR of GPX4, Col-2 and MMP-13 of the groups in this Figure (n = 5 for each group). **H** Quantification of Western blotting analysis for GPX4, Aggrecan, ADAMTS-5 and MMP-13 (n = 3 for each group). **I** DCFDA assays for ROS level and Mito-Tracker and JC-1 for mitochondrial membrane potential. Scale bar: 50 or 25 μm. **J** Quantitative analysis of mean fluorescence (n = 3 for each group). **K** The immunofluorescence of ATF6 and Col-2. Scale bar: 25 μm. **L** Quantitative analysis of mean fluorescence (n = 3 for each group). Results were shown as mean ± SEM. *ns* not significant, *p < 0.05, **p < 0.01, ***p < 0.001

Selenium supplementation was confirmed to enhance the expression of various selenoproteins besides GPX4 [36]. According to the above conclusions, relieving ER stress may have potential roles in alleviating mechanical overloading-induced ferroptosis in cells. SelK is a selenoprotein localized on ER, and it has been demonstrated that SelK gene knockout markedly enhanced ER stress in neurons [13, 37]. To explore the role of SelK in mechanical overloading-induced ER stress, we knocked down SelK using SiSelK in rat NP cells (Fig. 5A, B). Then we administered mechanical overloading on NP cells with Se-Met supplementation. The qPCR and Western blotting suggested upregulated SelK expression and decreased ATF6 and PERK in Se-Met supplementation group, while SiSelK group with Se-Met supplementation did not present these results (Fig. 5C–E). Interestingly, the expression of Calnexin was upregulated, and intracellular free Ca^2+^ level decreased with Se-Met supplementation, while the SiSelK group showed even the worst phenotype with Se-Met supplementation (Fig. 5D, F). Accordingly, the Se-Met supplementation group presented the highest expression of GPX4 (Fig. 5D, G, H). Moreover, the ROS synthesis was decreased, and mitochondrial dysfunction was improved after Se-Met supplementation, while the effects were absent when SelK was knocked down (Fig. 5I, J). Besides, the qPCR and Western blotting revealed that Se-Met supplementation reduced the production of ADAMTS-5 and MMP-13, and promoted the secretion of Aggrecan and Col-2, while knockdown of SelK aggravated ECM metabolic disorders (Fig. 5D, G, H). The Immunofluorescence for ATF6 and Col-2 indicated the same results as described above (Fig. 5K, L).

### Selenium supplementation alleviates the progression of IVDD in mice

**Fig. 6:**
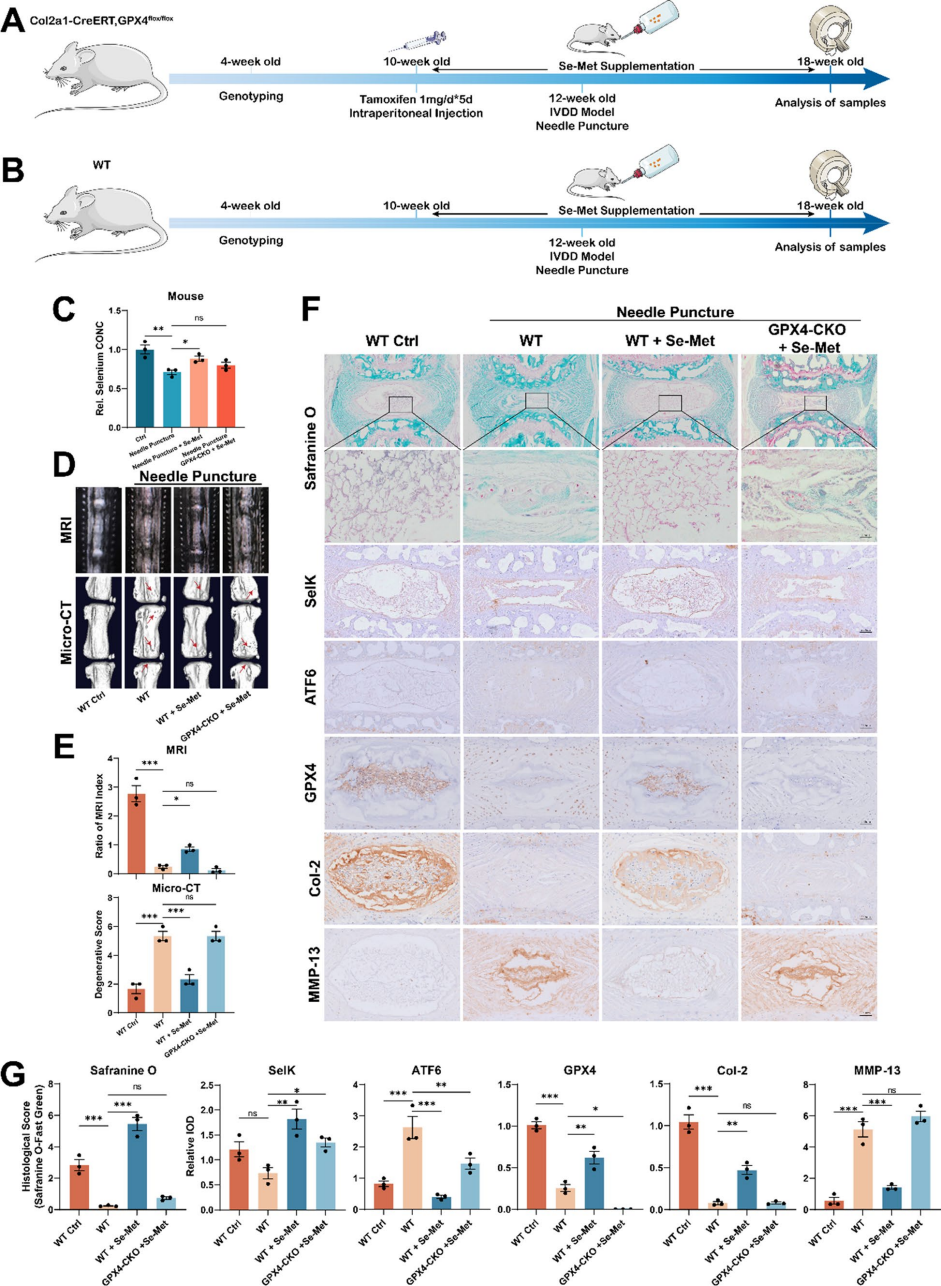
Selenium supplementation alleviates the progression of IVDD in mice. **A** Flowchart of animal experiment on GPX4-CKO mice. **B** Flowchart of animal experiment on WT mice. **C** The concentration of selenium of NP tissues from mice (n = 3 for each group). **D** The MRI and Micro-CT for coccygeal discs of mice. **E** Quantitative analysis of MRI and Micro-CT (n = 3 for each group). **F** The Safranine O-Fast Green staining and Immunohistochemical assay of SelK, ATF6, GPX4, Col2 and MMP-13. Scale bars: 50 or 250 μm. **G** Quantitative analysis of the Safranine O-Fast Green staining and Immunohistochemical assay (n = 3 for each group). Results were shown as mean ± SEM. *ns* not significant, *p < 0.05, **p < 0.01, ***p < 0.001

Here, WT and GPX4-CKO mice were used to verify the role of Selenium supplementation in vivo. Tamoxifen was administered to 10-week-old GPX4-CKO mice through intraperitoneal injection once a day for 5 days [38]. Thereafter, WT and GPX4-CKO mice were fed with water supplemented with Se-Met and maintained on the diet for 8 weeks throughout the experiments. And the IVDD model was established in 12-week-old WT and GPX4-CKO mice coccygeal discs. Six weeks after the establishment of the IVDD model, MRI and micro-CT for coccygeal discs were performed (Fig. 6A, B). The coccygeal disc tissues from the above mice were collected, and the concentration of selenium was detected. Interestingly, needle puncture reduced the concentration of selenium, but Se-Met supplementation improved this change, while this effect was attenuated in GPX4-CKO mice (Fig. 6C). The MRI and micro-CT revealed that Se-Met supplementation alleviated IVDD induced by needle puncture in WT mice. Nevertheless, with selenium supplementation, the phenotype of IVDD in GPX4-CKO mice was not improved (Fig. 6D, E). Furthermore, safranin-O staining was performed, suggesting the same trend results as described above (Fig. 6F, G). Immunohistochemical staining showed increased expression of SelK and decreased ATF6 in WT and GPX4-CKO mice with Se-Met supplementation (Fig. 6F, G). Moreover, increased expression of Col-2 and decreased expression of MMP-13 were observed in WT mice with Se-Met supplementation but not in GPX4-CKO mice (Fig. 6F, G), which indicated GPX4 was the key node at which Se-Met supplementation worked.

## Discussion

IVDD is a clinically common degenerative disease, and previous reports have shown that the disorder of NP cells and the destruction of NP tissue are critical features of IVDD [39, 40]. Static mechanical overloading damages the physiological functions of NP cells, which is reflected in an imbalance of anabolic and catabolic factors in IVD [6, 41, 42]. However, much about the underlying mechanisms involved in this process remains unknown.

Ferroptosis is a widespread pathophysiological cell death associated with mitochondrial dysfunction and cellular oxidative damage [43, 44]. It’s demonstrated that ferroptosis is involved in the pathogenesis of IVDD [45, 46]. In this study, compared to pfirrmann II IVD tissue, the ferroptosis marker protein GPX4 expression level significantly declined, and ACSL4 increased in pfirrmann V IVD tissue, implying a potential association between GPX4-regulated ferroptosis and IVDD development. In addition to the same results as described above, however, the microarray of the mechanical overloading model of NP cells also suggested the activation of ER stress pathways after mechanical stimulation. It was confirmed that ferroptosis and ER stress was observed simultaneously in both human and mouse degenerated IVD tissue by immunohistochemistry and Western blotting. So combined with the TEM images, we propose that mechanical overloading induces ferroptosis and ER stress in NP cells.

Calcium overload is accepted to promote ferroptotic damage to cells, and intracellular calcium homeostasis is managed by extracellular calcium influx and intracellular calcium outflow [47, 48]. It’s reported that activation of Piezo1 increased intracellular free Ca^2+^ level by inducing both intracellular calcium outflow through the endoplasmic reticulum localized Piezo1 ion channel as well as extracellular calcium influx through the plasma membrane-localized Piezo1 in a mechanism independent of the store-operated calcium entry [10, 49, 50]. Previous studies have found that mechanical overloading induced ferroptosis in chondrocytes by activating Piezo1 ion channel [8]. Here, we used SiPiezo1 and calcium-free medium with mechanical overloading and found that excessive mechanical stress induced ferroptosis and ER stress by increasing the intracellular free Ca^2+^ level through Piezo1 ion channel localized on plasma membrane and ER membrane. And interestingly, ER stress was attenuated after removing extracellular Ca^2+^ but stronger than when knock down the Piezo1 ion channel all over the cell, suggesting that elevated intracellular free Ca^2+^ level induced the activation of ER stress pathways, consistent with the results presented by Piezo1-CKO mice.

It’s reported that inhibition of ER stress could alleviate ferroptosis in colonic epithelial cells [33]. After removing extracellular Ca^2+^, we applied BIX while administering mechanical overloading on NP cells and found that the GPX4 expression was upregulated and the ECM metabolism disorder was relieved. Interestingly, intracellular free Ca^2+^ level was reduced and Ca^2+^ level in the ER was elevated after inhibition of ER stress during the above process. We, therefore, propose that intracellular free Ca^2+^ level elevation and the activation of ER stress are positive feedback processes that promote each other.

According to the above conclusion, relieving ER stress is important to alleviate mechanical overloading-induced ferroptosis in NP cells. SelK is a selenoprotein localized on ER, and it has been demonstrated that SelK gene knockout markedly enhanced ER stress in neurons [13, 51]. Selenium, as an essential micronutrient, has been confirmed that selenium supplementation could enhance the expression of various selenoproteins in cells [52, 53]. So we administered mechanical overloading on NP cells along with Se-Met supplementation. Surprisingly, SelK expression was upregulated, and the intensity of ER stress was diminished, decreasing intracellular free Ca^2+^ level and improving ECM metabolism disorders. As a control experiment, we provided the same regimen of Se-Met supplementation to SiSelK NP cells, but no enhancement to SelK or relieving ER stress was observed. Thus, SelK might be a key regulator in mechanical overloading-induced ER stress in NP cells.

GPX4 is a pivotal selenoprotein of ferroptosis, being protective against ferroptotic damage in cells under various conditions [54, 55]. Selenium-GPX4 axis was proven to protect follicular helper T cells from ferroptosis [16]. Mechanical overloading is reported to interfere with the normal production and function of GPX4 by increasing intracellular free Ca^2+^ level in chondrocytes [8]. In this study, mechanical overloading increased intracellular free Ca^2+^ level through calcium influx and outflow, leading to a decrease in GPX4 expression in NP cells. Excitingly, GPX4 expression was upregulated, and mitochondrial dysfunction and oxidative stress were alleviated; eventually, the ECM metabolism homeostasis was improved after selenium supplementation. Moreover, GPX4-CKO mice displayed poorer intervertebral disc ECM phenotype compared with WT mice of the same age, implying that GPX4 plays an important role in the normal ECM metabolism of the intervertebral disc. Interestingly, after selenium supplementation around the establishment of the IVD needle puncture model for 8 weeks, the selenium-supplementation group showed improved performance of ferroptosis and ER stress and better ECM metabolism phenotype compared to the puncture group. Notably, selenium supplementation failed to relieve ferroptosis in GPX4-CKO mice, suggesting that GXP4 expression in NP cells is necessary to mediate the benefit of selenium supplementation.

**Fig. 7:**
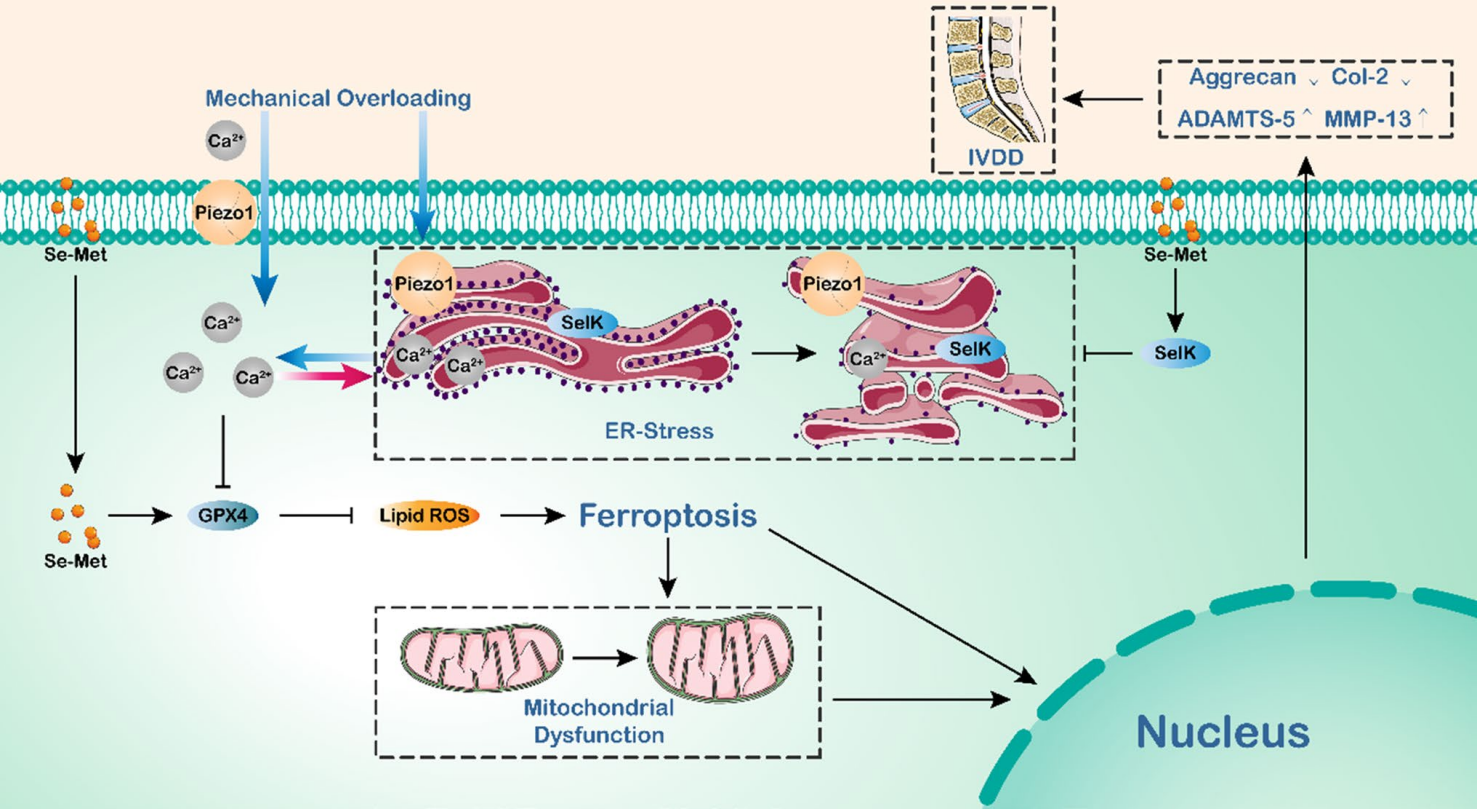
Schematic diagram of Selenium-SelK-GPX4 axis protects nucleus pulposus cells from mechanical overloading-induced ferroptosis

Taken together, our data propose that in NP cells, when the Piezo1 ion channel localized on the plasma membrane and ER membrane is activated by mechanical overloading, extracellular calcium influx and intracellular calcium outflow are strengthened, leading to intracellular calcium overload, which result in ER stress, further aggravating ER calcium outflow, and then the production and function of GPX4 is obstructed, inducing ferroptosis and ECM metabolism disorders. However, selenium supplementation could upregulate SelK expression, alleviate ER stress, and enhance GPX4 expression, attenuate cellular oxidative stress and ferroptosis. In summary, ferroptosis plays an important role in mechanical overloading-induced IVDD, and selenium supplementation promotes significance to attenuate ferroptosis and thus alleviate IVDD, which might provide insights into potential therapeutic interventions for IVDD (Fig. 7).

## Materials and methods

### Human tissue specimens

Human NP specimens were obtained from patients who underwent lumbar spine surgery (May 2019–October 2021) at Qilu Hospital of Shandong University. The relatively normal human lumbar NP tissues were obtained from 5 patients (age ranges 16–36 years, grade I ~ II) with acute lumbar disc herniation or adolescent idiopathic scoliosis but without IDD. The degenerated NP were obtained from 10 patients (age ranges 45–70 years, grade III ~ V) with IVDD. The degenerated degree of IDD was assessed by Pfirrmann grading system [18]. All study procedures were approved by the Medical Ethical Committee of Qilu Hospital of Shandong University. The patients involved in this study signed informed consent documents and voluntarily agreed to participate in this research.

### Animals

All animal experiments in this study were performed in accordance with the International Guiding Principles for Animal Research and were approved by the Laboratory Animal Centre of Shandong University. Col2a1-CreERT mice were established by and purchased from Cyagen (USA). GPX4^flox/+^ mice were created by Cyagen (USA) through ES genome engineering. GPX4^flox/+^ mice were mated with GPX4^flox/+^ mice to generate GPX4^flox/flox^ mice. GPX4^flox/flox^ mice were mated with Col2a1-CreERT mice to generate Col2a1-CreERT, GPX4^flox/+^ mice. Col2a1-CreERT GPX4^flox/+^ mice were mated with Col2a1-CreERT GPX4^flox/+^ mice to generate Col2a1-CreERT GPX4^flox/flox^ mice. Male mice with the Col2a1-CreERT and GPX4^flox/flox^ genes were used in experiments. Ten-week-old Col2a1-CreERT GPX4^flox/flox^ mice were intraperitoneally injected with tamoxifen (1 mg/d*5 d) (HY-13757A, MCE, USA) to obtain GPX4-conditional knockout (GPX4-CKO) mice. Piezo1^flox/+^ mice were created by Cyagen (USA) through ES genome engineering. Piezo1^flox/+^ mice were mated with Piezo1^flox/+^ mice to generate Piezo1^flox/flox^ mice. Piezo1^flox/flox^ mice were mated with Col2a1-CreERT mice to generate Col2a1-CreERT, Piezo1^flox/+^ mice. Col2a1-CreERT Piezo1^flox/+^ mice were mated with Col2a1-CreERT Piezo1^flox/+^ mice to generate Col2a1-CreERT Piezo1^flox/flox^ mice. Male mice with the Col2a1-CreERT and Piezo1^flox/flox^ genes were used in experiments. Ten-week-old Col2a1-CreERT Piezo1^flox/flox^ mice were intraperitoneally injected with tamoxifen (1 mg/d*5 d) (HY-13757A, MCE, USA) to obtain Piezo1-conditional knockout (Piezo1-CKO) mice. Col2a1-CreERT GPX4^+/+^ Piezo1^+/+^ littermates were assigned to the wild-type (WT) group. Three-month-old Sprague–Dawley (SD) rats were purchased from the Animal Center of Shandong University. All of the animals were housed under controlled identical specific pathogen-free (SPF) standard environmental conditions (23 ± 2 ℃, 12 h light/dark cycle) with free access to food and allowed to move freely. WT and GPX4-CKO mice were fed with water supplemented with Se-Met (2 mg/L, HY-114245, MCE, USA) and maintained on the diet for 8 weeks around establishment of IVD needle puncture model.

### Genotyping

Tail clippings were obtained from 4-week-old mice. Mouse tail DNA was extracted using a One Step Mouse Genotyping Kit (PD101-01, Vazyme, China) according to the manufacturer’s instructions. Agarose (1.5 g), 100 mL of 2 * Tris–acetate-EDTA buffer (TAE) and 6 ul of Gel Red were mixed and heated to form agarose gels. The amplified DNA was subjected to agarose gel electrophoresis. Images were captured using an Amersham Imager 680 (GE, USA). The primers used for amplification (GPX4flox, Piezo1flox and Col2a1-CreERT) are listed in Table 1.

**Table 1:** Primers used for gene identification

Target	Forward primers, 5ʹ–3ʹ	Reverse primers, 5ʹ–3ʹ
GPX4flox	TCCATTGGTCGGCTGCGTGAGG	ACCCTGGATACGGTGACCCGAC
Piezo1flox	AGCAAGGCCAATGTAGTATCTGG	CTATTGGTGCCTAGTTGGCAGAC
Col2a1-CreERT	CACTGCGGGCTCTACTTCAT	ACCAGCAGCACTTTTGGAAG

### IVDD model establishment

The caudal needle puncture injuries were performed in 12 weeks old WT mice (n = 20) and GPX4-CKO mice (n = 10) to establish IVDD model in vivo. The surgeries were performed under general anesthesia (2% isoflurane in oxygen) and sterile conditions. After confirming the location of IVD using microscope, needle punctures were created using 26 G syringe needles to a depth of 50% of dorsal–ventral width [56]. Mice were closely monitored to ensure the absence of operative complications and allowed free activity in their cages with access to food and water.

### Isolation and culture of rat primary nucleus pulposus cells

Rat Primary Nucleus Pulposus Cells were extracted as previously described [57, 58]. Briefly, we isolated nucleus pulposus tissues from lumbar discs of Sprague–Dawley rats and the cells were digested with 0.2% collagenase type II (Gibco, USA) at 37 ℃ for 8 h. The cells were then cultured in DMEM/F12 (HyClone, USA) supplemented with 10% foetal bovine serum (FBS, Gibco, USA), 1% penicillin and streptomycin (P1400, SolarBio, China) under standard incubation conditions (37 ℃, 5% CO2). The culture medium was replaced every 3 days, and the cells were passaged when they reached 80–90% confuence. The cells from within five generations were used in all vitro experiments.

### In vitro mechanical stress culture model

We used an in vitro mechanical stress culture model designed in the previous study of our research group, which is similar to the device described by others [8, 59, 60]. The cells were preplanted on a 14- or 24-mm cell slide, which was placed on the scaffold. Cell slides and scaffolds were placed in a closed chamber filled with complete culture medium. A deformable rubber sheet above the closed chamber was applied to pressurize the inside of the liquid. The cells were exposed to mechanical stress at 1 MPa with a frequency of 1 Hz for 1 h by a pneumatic component (FESTO, Germany) according to the research of others. Before the experiment, we placed the medium in the closed chamber into the cell incubator for 6 h to maintain cell viability. Depending on the experimental conditions, medium (DMEM/F12 (HyClone, USA) and medium without calcium ions (DMEM/F12 without CaCl (Basalmedia, China)) were used. And the following drugs were used: 5 μM Bip Inducer X (HY-110188, MCE, USA), 10 μM BAPTA-AM (HY-100545, MCE, USA), 5 μM Se-Met (HY-114245, MCE, USA), 10 μM ML210 (HY-100003, MCE, USA). At the end of the pressure application, the cells or the femoral head were placed in new complete medium.

### Microarray RNA-sequence

Rat NP cells were stimulated as indicated. After 24 h, cells were harvested by a cell brush and then centrifuged. Then cell precipitates were collected, quick-frozen in liquid nitrogen, and transported on dry ice. Overall gene expression was examined by Qinglian Biotech (Beijing, China).

### Transmission electron microscopy (TEM)

Rat NP cells were stimulated as indicated. After 24 h, cells were harvested and TEM fixative was added at 4 ℃ for preservation and transportation. The images were taken by Servicebio (Wuhan, China).

### Safranine O staining

Safranine O staining was performed to detect the changes in proteoglycans with a Safranine O staining kit (G1371, SolarBio) according to the manufacturer’s recommended procedure.

### Immunohistochemistry

After fixation in 4% paraformaldehyde, the IVD tissues were decalcified, embedded in paraffin, and cut into 5-μm sections. After the paraffin sections were dewaxed with xylene and gradient ethanol, they were antigen-repaired with citric acid (pH 6.0) and blocked with goat serum. Then the sections were incubated with primary antibodies against GPX4 (1:1000, Proteintech), ACSL4 (1:200, Proteintech), ATF6 (1:200, Proteintech), Piezo1 (1:200, Affinity), SelK (1:200, Affinity), Col-2 (1:200, Novus), MMP-13 (1:200, Proteintech) at 4 ℃ overnight. The next day, the sections were incubated with goat anti-rabbit IgG-HRP secondary antibody for 1 h at room temperature. Detection was performed by using the DAB Substrate kit (ZLI-9018, ZSGB) and were counterstained with 1% hematoxylin. Images were captured by a microscope (Leica DMI3000B). The positive areas were quantified by ImageJ.

### Total protein extraction and western blotting

The collected NP tissues was ground to powder in liquid nitrogen. The cells at 24 h after treated with mechanical stress or above tissues powder were placed in RIPA lysis buffer (Beyotime, China) supplemented with 1 mM PMSF (Beyotime, China) on ice for 30 min. The collected liquid was centrifuged at 12,000 rpm for 15 min at 4 ℃. The protein concentration was detected with a BCA protein assay kit (PC0020, Solarbio). The protein samples from each group were separated in 8%, 10%, or 12% SDS–polyacrylamide gels (SDS-PAGE) and then transferred to a polyvinylidene difluoride (PVDF) membrane (Millipore, USA). After blocking with QuickBlockTM Blocking Buffer (Beyotime, China) for 20 min at room temperature, the membranes were incubated with rabbit primary antibodies against ACSL4 (1:2000, Proteintech), Bip (1:2000, Proteintech), Piezo1 (1:1000, Proteintech), Calnexin (1:5000, Proteintech), GPX4 (1:1000, Proteintech), ATF6 (1:2000, Proteintech), PERK (1:1000, Proteintech), Aggrecan (1:1000, Proteintech), Col-2 (1:1000, Novus), ADAMTS-5 (1:1000, Abcam), MMP-13 (1:1000, Proteintech), SelK (1:500, Proteintech), GAPDH (1:5000, Proteintech) overnight at 4 ℃. Then, the membranes were incubated for 90 min at room temperature with secondary antibody. The bands were visualized using an Amersham Imager 600, and the density was quantified using ImageJ software.

### RNA extraction and qPCR

An RNAfast200 Kit (220011, Fastagen) was used to extract total RNA from the NP cells according to the recommended procedure. Total RNA (1 µg) was reverse-transcribed to complementary DNA (cDNA) using HiScript II Q RT SuperMix for qPCR (R222-01, Vazyme). The qPCR was carried out with RealStar Fast SYBR qPCR Mix (A301, GenStar). The experiment was repeated five times for each target gene of each group. The nucleotide sequences of the primers are listed in Table 2. The expression levels of target genes were normalized to GAPDH and were calculated by the 2 − ΔΔCT method.

**Table 2:** Primers used for qPCR

Target	Forward primers, 5ʹ–3ʹ	Reverse primers, 5ʹ–3ʹ
ACSL4	TGTGAGCGCATACCTGGATT	CAGCCGTAGGTAAAGCAGGA
Bip	GGACCACCTATTCCTGCGTC	CAATCAGACGCTCCCCTTCA
Calnexin	AATAGAATGCGGTGGTGCCT	TGGGGTTTTTGTGGCGAAAG
ATF6	AGGTGAAGACTGGGAGTCCAC	ACTCCCAAGGCATCAAATCCA
PERK	GATACGGCATTTGGCTTGGG	AGTTCCACGTCGTCATCGG
GPX4	CTCGCAATGAGGCAAAACCG	GGGAAGGCCAGGATTCGTAA
Col-2	TTTGACGAGAAGGCTGGTGG	GGACCAATGGGACCAGAGAC
MMP-13	ACCCAGCCCTATCCCTTGAT	TCTCGGGATGGATGCTCGTA
Aggrecan	CTGAATGGGAGCCAGCCTAC	GATGTGGAAGGGACTTGCGA
ADAMTS-5	TTCACGCATCCTGCATGTCT	TGTGCGTCGCCTAGAACTAC
SelK	GTCCCCCTGGAGATTGTCTTT	TTCGTGGAGGGTTTCCTGGT
GAPDH	TCTCTGCTCCTCCCTGTTCT	ATCCGTTCACACCGACCTTC

### Calcium imaging

The NP cells were loaded with Fura-2 AM (Yeasen, China) at 37 ℃ with stress apply and fluorescence intensity of intracellular Fura-2-AM was measured at two distinct wavelengths (ex 340/em 515 and ex 380/em 515) to assess bound and unbound states according to the manufacturer’s instruction.

### Reactive Oxygen Species Assay

To detect intracellular reactive oxygen species (ROS), we used an ROS assay kit (S0033, Beyotime). All the procedures were performed according to the manufacturer’s instructions. Briefly, after washing twice with sterile PBS, cells were stained with 10 μM DCFDA at 37 ℃ for 20 min in the dark. Then, the cells were washed with basal culture medium three times. The images were captured using a fuorescence microscope (ZEISS Vert. A1).

### MitoTracker Assay

MitoTracker Red CMXRos (C1049B, Beyotime) was used to detect mitochondrial activity. According to the manufacturer’s instructions, the cells were incubated with a culture medium containing 20 nM MitoTracker Red CMXRos for 30 min at 37 ℃ in the dark and then observed and captured with a fluorescence microscope (ZEISS Vert. A1) after changing the fresh culture medium.

### JC-1 Assay

The mitochondrial membrane potential changes were detected with a JC-1 assay kit (C2006, Beyotime). Based on the manufacturer’s instructions, the cells were stained with the JC-1 staining solution at 37 ℃ for 20 min free from light. Then, the cells were washed twice with JC-1 staining buffer, and the images were observed and captured using a fuorescence microscope (ZEISS Vert. A1).

### Immunofluorescence staining

The cells were treated as indicated, and after 48 h, the cells were fixed with 4% paraformaldehyde for 20 min. After being permeabilized with 0.2% TritonX-100 for 20 min, the samples were blocked by BSA at 37 ℃ for 1 h. Then, the cells were incubated with antibodies against ATF6 (1:100, Proteintech) and Col-2 (1:500, Novus) at 4 ℃ overnight. The next day, the cells were incubated with fuorescently labelled goat anti-rabbit or anti-mouse IgG (1:100, Abbkine) for 1 h at 37 ℃. The nuclei were stained with DAPI. The images were taken using a fuorescence microscope (ZEISS Vert. A1) and analysed with the ImageJ software.

### Selenium concentration examination

To examine the concentration of selenium of NP tissues and cells, a Selenium Assay Kit (abx298910, abbexa) was used. All procedures were performed according to the manufacturer’s instructions with a luminometer (Centro XS3 LB 960, Berthold Technologies).

### In vitro siRNA transfection

Small interfering RNAs were constructed by GenePharma and used to inhibit the expression of Piezo1 and SelK (Table 3). Rat NP cells were cultured in six-well plates to 60–70% confluence and were transfected with 50 nM negative control (NC), Piezo1 or SelK siRNA using Lipofectamine 2000 (ThermoFisher) according to the manufacturer’s instructions. After 48 h, cellular lysates were obtained to analyze the expression of the genes of interest.

**Table 3:** Sequences of SiSelKs

SiSelK	Forward Primers, 5ʹ–3ʹ	Reverse Primers, 5ʹ–3ʹ
Si-1	CUCGAAUGGUCAGGUGUUATT	UAACACCUGACCAUUCGAGTT
Si-2	GAAUAGCAGAAUUUGUGGUTT	ACCACAAAUUCUGCUAUUCTT
Si-3	GAUGUGCAGUUCUAUAAAUTT	AUUUAUAGAACUGCACAUCTT

### Magnetic resonance imaging (MRI)

To evaluate the structural differences and signal intensity changes in sagittal T2-weighted images of IVDs, the mice underwent MRI scanning after the initial puncture 6 weeks. Disc imaging examination was performed using 3.0 T MRI scanners (GE Signa HDX, USA). Mice were kept in the supine position with the tail in straightened state. The specific parameters included spin echo repetition time, 2275 ms; echo time, 80 ms; number of excitations, 8; field of view, 5 cm; slice thickness, 1.5 mm; no phase wrap. The calculation of T2 intensities and MRI indexes (the area of NP multiplied by the average signal intensity) followed the methods described in a previous study [61].

### Micro-CT

The scanning protocol included an isometric resolution of 15 μm, with X-ray energy settings of 70 kV and 200 μA. The microstructure of the vertebrae was measured through a Quantum GX2 scanner (PerkinElmer, USA). Prior to histological processing, samples were fixed in paraformaldehyde and used for micro-CT. The scanned images from each group were evaluated at the same threshold to allow 3-dimensional structural reconstruction of each sample. The degenerative score followed the methods described in a previous study [62].

### Statistical analyses

Analysis of data was performed with GraphPad Prism (GraphPad Software Inc., USA). Comparisons of various groups were performed using analysis of variance (ANOVA) with Tukey’s post hoc test. Data were presented as “mean ± SEM”. Statistical signifcance was indicated when p < 0.05.

### Supplementary Information

Below is the link to the electronic supplementary material.

Supplementary file1 (DOCX 7266 KB)

## Data Availability

The datasets generated and/or analyzed during the current study are available from the corresponding authors on reasonable request.
