# Supplementary material for: Selenium-SelK-GPX4 axis protects nucleus pulposus cells against mechanical overloading-induced ferroptosis and attenuates senescence of intervertebral disc
Source: Cell Mol Life Sci. 2024 Jan 22;81(1):49. doi: 10.1007/s00018-023-05067-1 (PMC10803455; doi:10.1007/s00018-023-05067-1)
Supplement: Supplementary file 1 — Supplementary file1 (DOCX 7266 KB) [file 18_2023_5067_MOESM1_ESM.docx]

**Supplementary Materials**

Selenium-SelK-GPX4 axis protects nucleus pulposus cells against mechanical overloading-induced ferroptosis and attenuates senescence of intervertebral disc

Chunwang Jia^1, #^, Ziqian Xiang^1, #^, Pengfei Zhang^1, #^, Long Liu^2^, Xuetao Zhu^1^, Ruixuan Yu^1^, Zhicheng Liu^1^, Shaoyi Wang^1^, Kaiwen Liu^1^, Zihao Wang^1^, Krasimir Vasilev^3, 4^, Shuanhu Zhou^5^, Ziwen Geng^6^, Xinyu Liu^1^, Yunpeng Zhao^1, *^, Yuan Gao^1, *^, Lei Cheng^1, *^, Yuhua Li^1, *^

This supporting information includes **Supplementary Fig. S1-S7**

**Supplementary Figures**

**
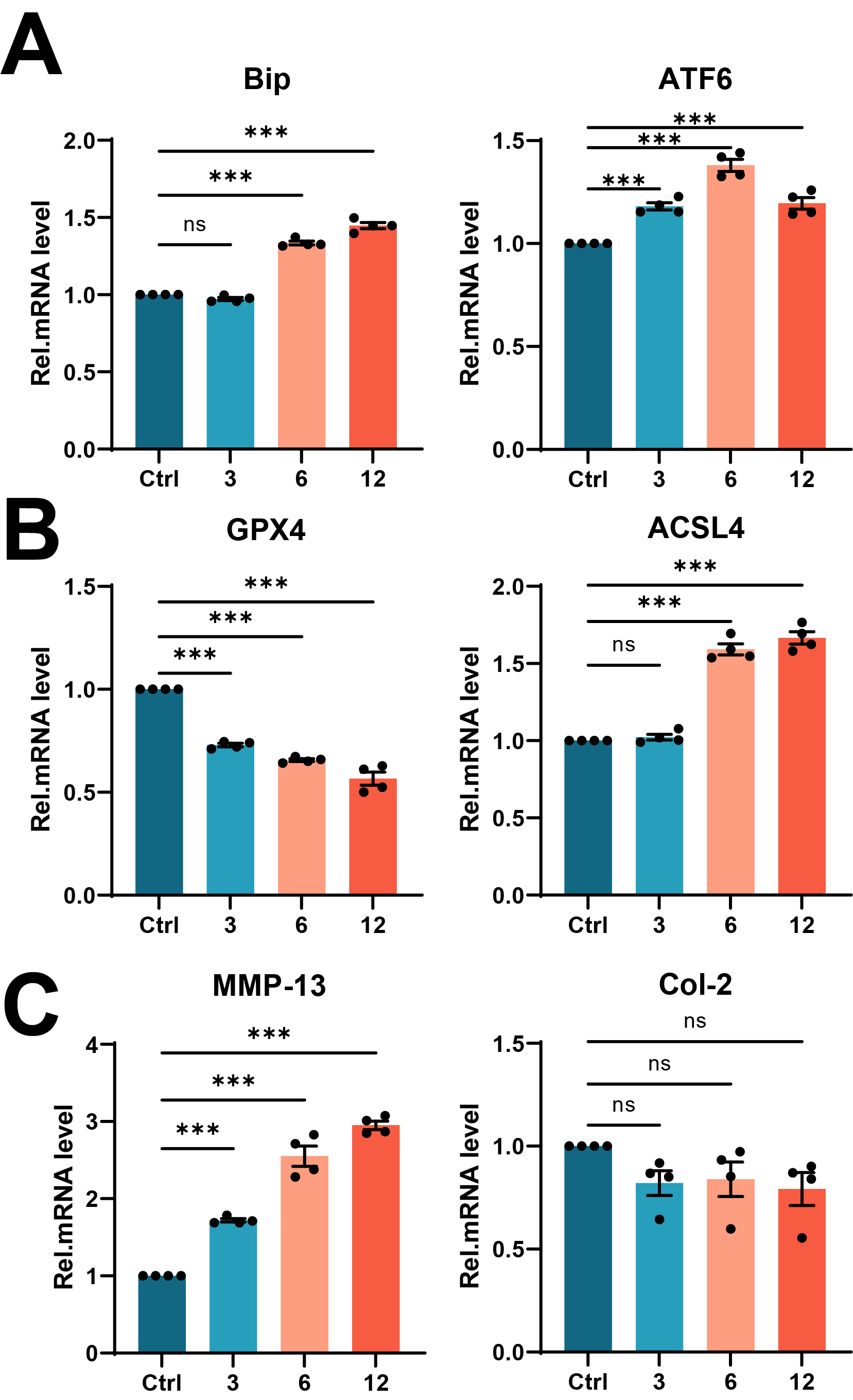
**

**Supplementary Fig. S1** The total mRNA was extracted at 3h, 6h and 12h after stress apply and analyzed by qPCR for Bip, ATF6, ACSL4, GPX4, MMP-13 and Col-2 (n = 4 for each group).


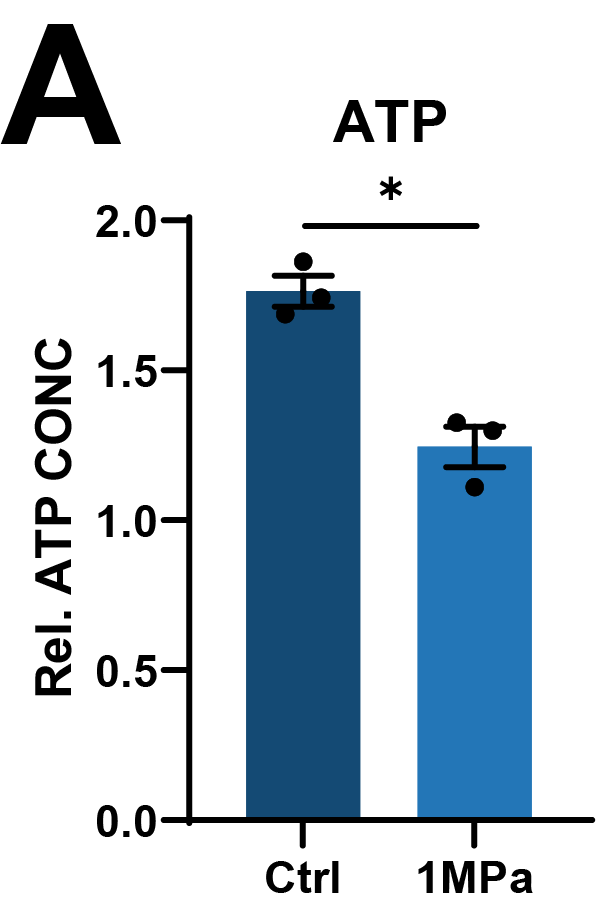


**Supplementary** **Fig. S2** ATP levels in each indicated group were examined.


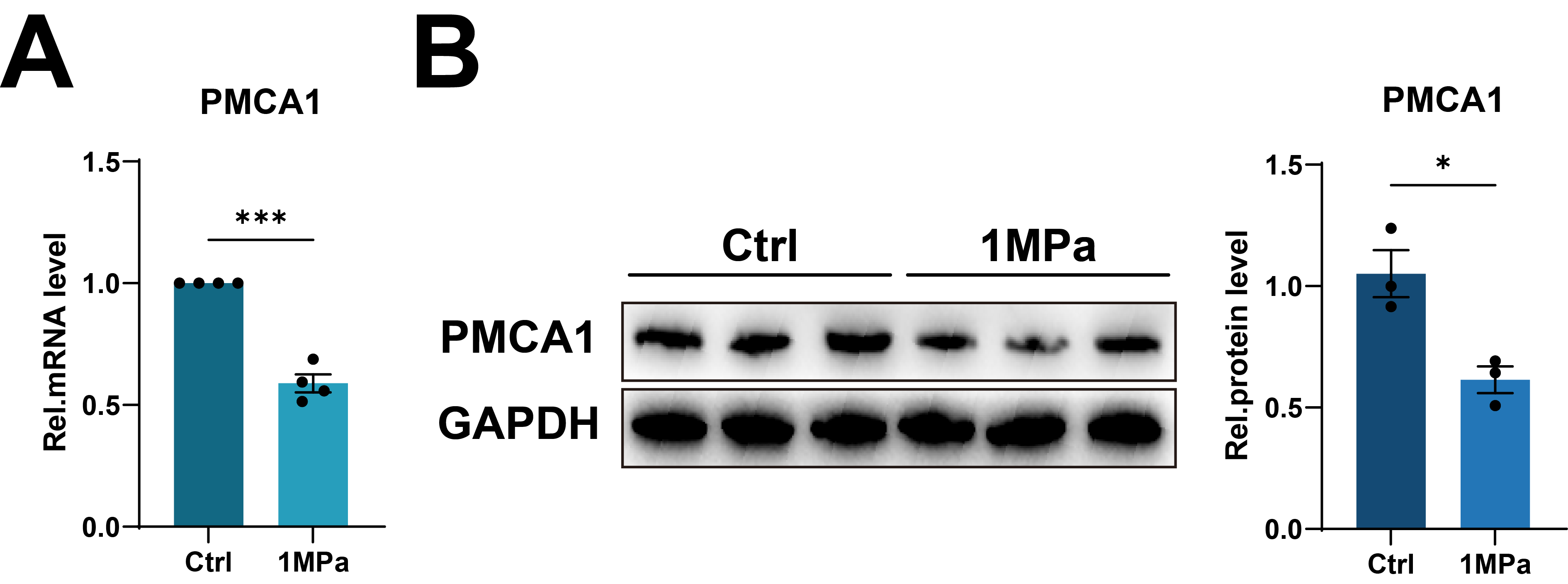


**Supplementary Fig. S3** The qPCR (n = 4 for each group) and Western blotting analysis (n = 3 for each group) of PMCA1 of the groups in this Figure.


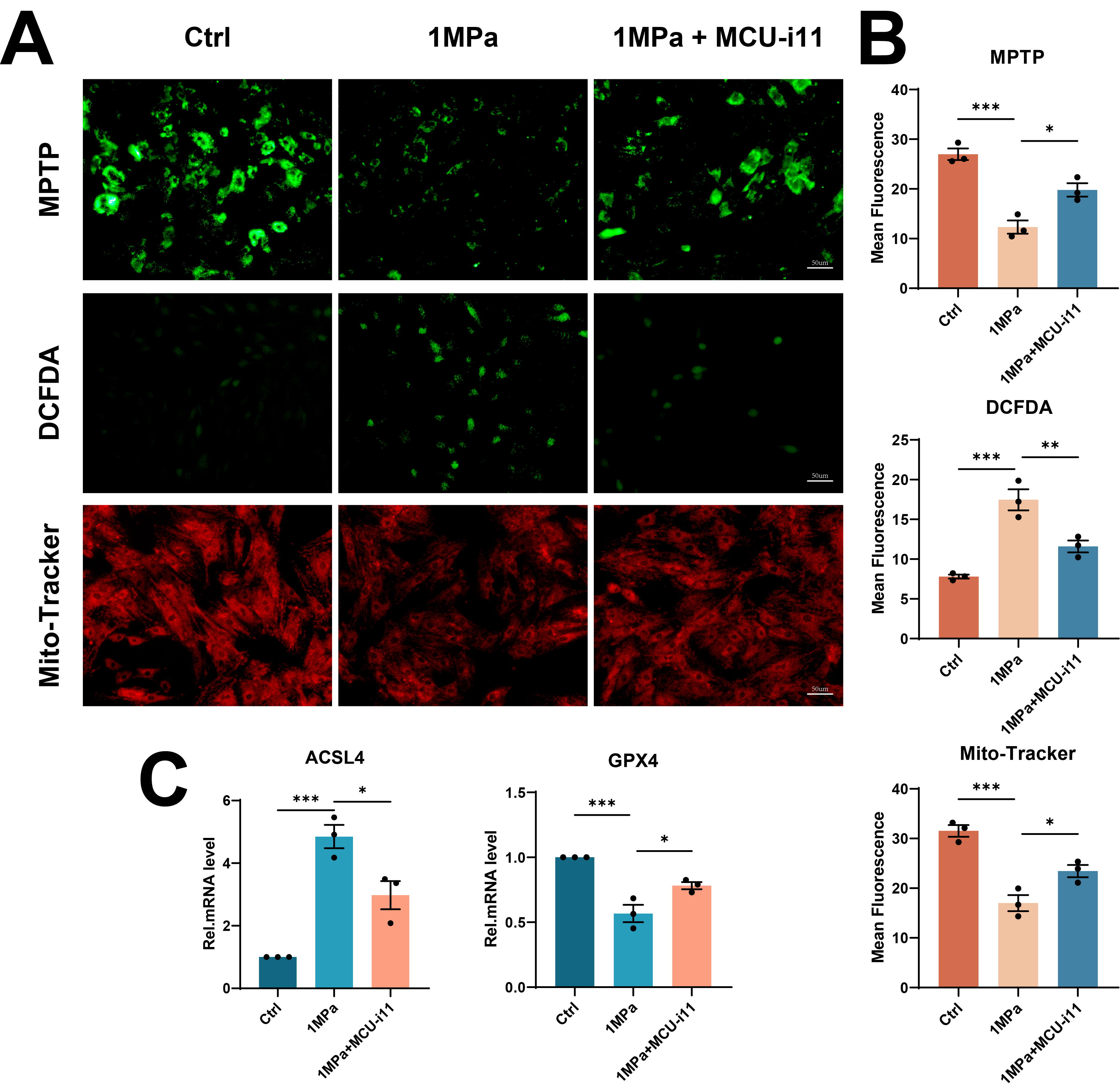


**Supplementary Fig. S4** (A-B) MPTP assays, DCFDA assays for ROS level and Mito-Tracker for mitochondrial membrane potential. Scale bar: 50 μm. (C) The qPCR of ACSL4 and GPX4 (n = 3 for each group) of the groups.


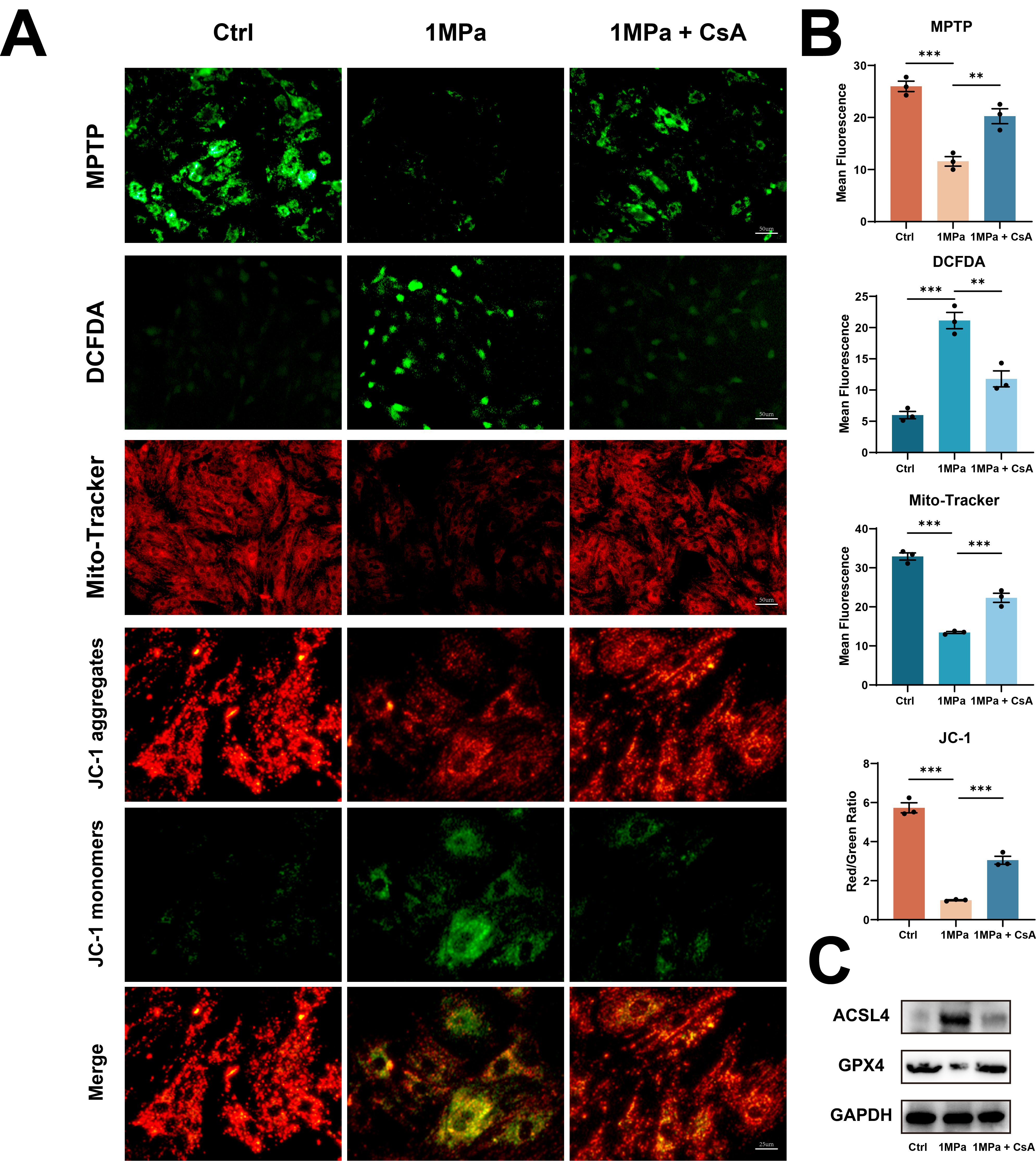


**Supplementary Fig. S5** (A-B) MPTP assays, DCFDA assays for ROS level and Mito-Tracker and JC-1 for mitochondrial membrane potential. Scale bar: 50 or 25 μm. (C) Western blotting analysis (n = 3 for each group) of ACSL4 and GPX4 of the groups.


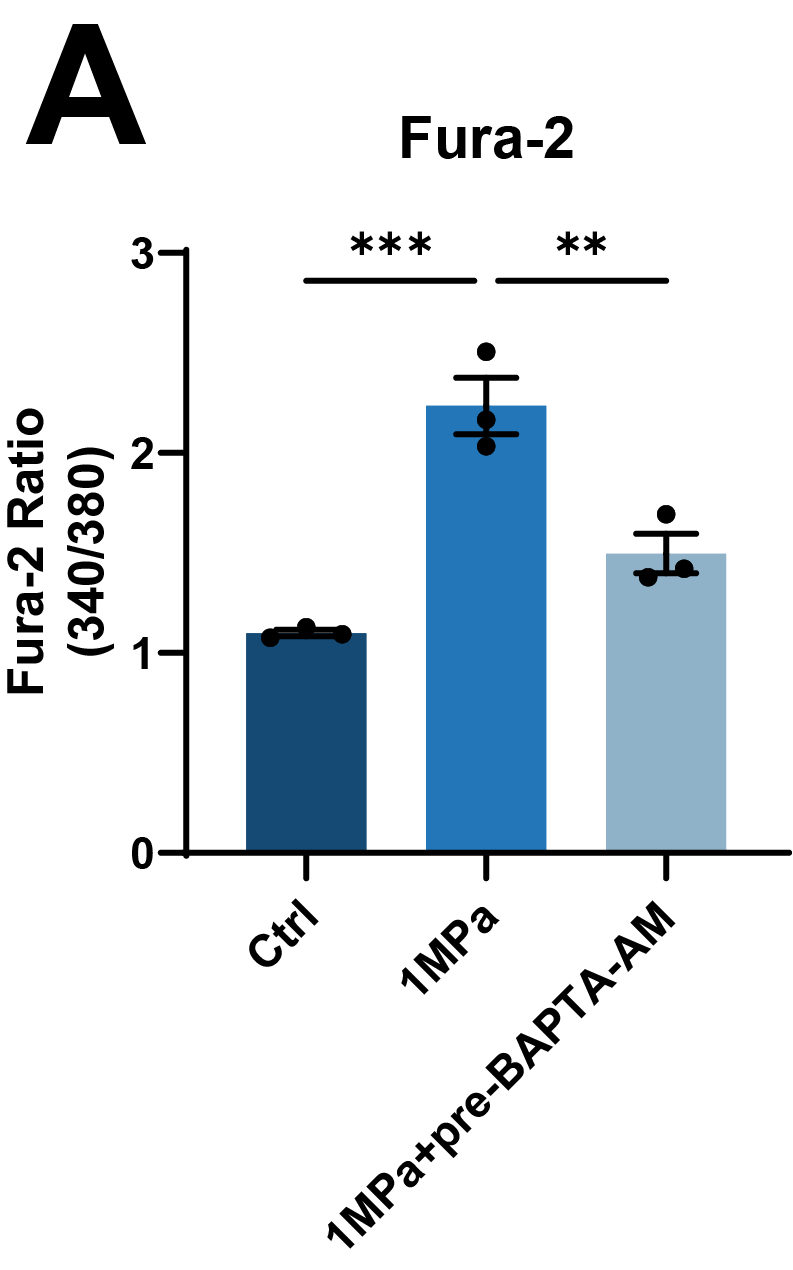


**Supplementary** **Fig. S6** The Fura-2 AM to detect intracellular free Ca^2+^ level (n = 3 for each group).


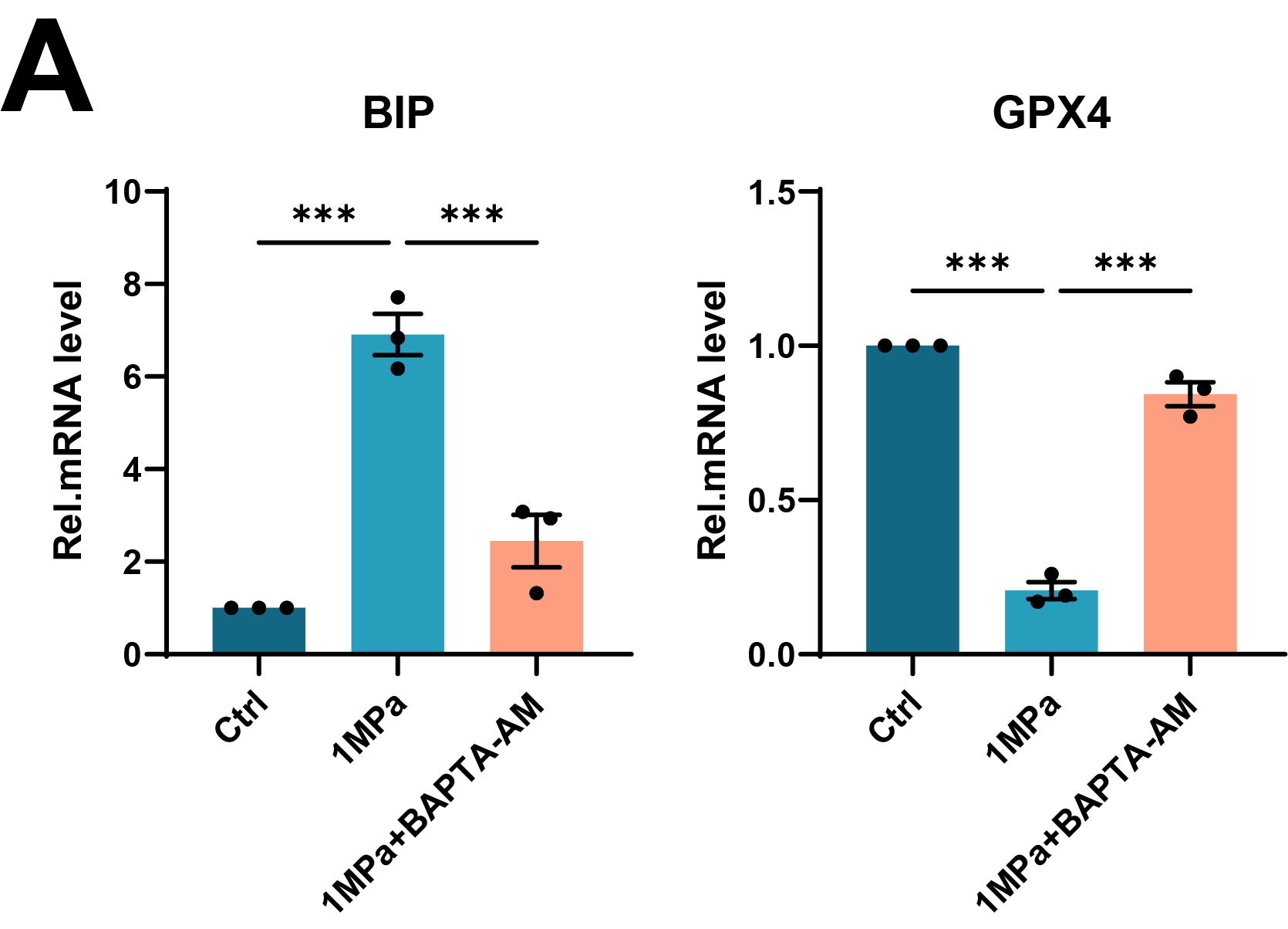


**Supplementary** **Fig. S7** The qPCR of Bip and GPX4 (n = 3 for each group).
